# Mining Migrant Worker Recruitment Policy and the Production of a Silicosis Epidemic in Late 20th-Century Southern Africa

**DOI:** 10.5334/aogh.4059

**Published:** 2023-03-27

**Authors:** Rodney Ehrlich, Stephen Barker, Alex Montgomery, Peter Lewis, Barry Kistnasamy, Annalee Yassi

**Affiliations:** 1 Division of Occupational Medicine, School of Public Health and Family Medicine, University of Cape Town, Cape Town 7925, ZA; 2 Global Health Research Program, School of Population and Public Health, University of British Columbia, CA; 3 DataFirst, University of Cape Town, Rondebosch, Cape Town 7700, ZA; 4 Independent Consultant in Labour Relations and Labour History, Cape Town, ZA; 5 Compensation Commissioner for Occupational Diseases, Johannesburg, ZA

**Keywords:** silicosis, mining, migrants, South Africa, labour policy

## Abstract

**Objectives:**

Between the 1980s and 2000s, an epidemic of silicosis was identified in migrant black gold miners, many from neighbouring countries, who had worked in the South African gold mines. This study uses the newly available employment database of a large gold mining company to demonstrate how a sustained rise in employment duration in a new cohort of black migrant workers resulted from changes in recruitment policy, and it examines the implications for current surveillance and redress.

**Methods:**

Contract data of 300,774 workers from the employment database of a multi-mine gold mining company were analysed for 1973–2018. Piecewise linear regression was applied to determine trends in cumulative employment, including South African versus cross-border miners. The proportions with cumulative employment of at least 10, 15, or 20 years, typical thresholds for chronic silicosis, were also calculated.

**Results:**

Five calendar phases were identified between 1973 and 2018. During the second phase, 1985–2013, mean cumulative duration of employment rose fivefold, from 4 to 20 years. Cumulative employment continued to rise, although more slowly, before peaking in 2014 at 23.5 years and falling thereafter to 20.1 years in 2018. Over most of the 1973–2018 period, miners from neighbouring countries had greater cumulative employment than South African miners. Overall, the proportion of miners exiting with at least 15 years of cumulative employment rose from 5% in 1988 to 75% in 2018. This report identifies a number of fundamental changes in labour recruitment policy in the gold mining industry in the 1970s which provide an explanation for the subsequent rise in cumulative exposure and associated silicosis risk.

**Conclusions:**

These new data support the hypothesis of a silicosis epidemic driven by increasing cumulative silica dust exposure in a new cohort of circular migrant workers from the 1970s. They inform current programmes to improve surveillance of this neglected population for silicosis and related disease and to provide medical examinations and compensation to a large number of former gold mines. The analysis highlights the lack of information on cumulative employment and silicosis risk among migrant miners in previous decades. The findings have global relevance to the plight of such migrant workers in hazardous occupations.

## Introduction

From the beginning of the 20th century, a central feature of the South African gold mining industry has been a system of circular migrant labour between the rural areas of Southern Africa and the mines, to which workers were recruited from both South Africa and neighbouring countries [1,2,3,4]. This system was closely tied to the occupational colour bar, by which migrant black workers came to fill the large majority of underground labouring occupations, while supervisory, blasting, and skilled artisanal and technical jobs were mostly the reserve of white workers. The last elements of the colour bar, notably the restriction of blasting certificates to white miners, were only removed from statute in 1987 [1].

By the late 20th and early 21st centuries, a triple epidemic of silicosis, tuberculosis, (TB), and HIV infection had become apparent in this black migrant worker population [4,5]. These diseases, associated with occupation and migrancy, are closely interlinked [4,5,6,7], imposing a disproportionately heavy burden on migrant workers. Silicosis is a chronic fibrotic lung disease caused by exposure to respirable silica dust, a component of gold-bearing ore released in the process of drilling, blasting, and ore transport. Both silica exposure and silicosis are strong risk factors for pulmonary TB, adding further risk in a population with already elevated rates of TB [4,5,6]. The southern African HIV epidemic, itself linked to the circular migrant labour system and single-sex accommodation on the mines, has been a potent amplifier of TB in combination with silicosis [7].

Between 1973 and 2007, South Africa’s statutory mining autopsy system revealed a tenfold increase in the proportion of autopsies (for non-natural causes of death) showing evidence of silicosis in black miners [8]. Between 1984 and the 2000s, workforce surveys showed a fourfold increase in crude silicosis prevalence associated with a rise in the proportion of workers aged > 45 years from 5.8% in the first study [9] to 43.6% in the second [10]. Other surveys of older and/or long-service miners and ex-miners in the 1990s and early 2000s recorded prevalences of silicosis ranging from 19% to 36% [5,11,12].

The past three decades have seen a number of research and advocacy efforts to uncover this burden of disease and to secure compensation for these workers [11,13,14,15]. Most recently, after a century of neglect of migrant ex-miners by the mining industry and the state, two major compensation programmes are in process. The first is the rehabilitation of the statutory compensation system covering lung disease in miners [16,17]. The second is the settlement of a class-action suit for silicosis and TB for 5 billion rand (US $273 million as of February 23, 2023), resulting in the formation of the civil Tshiamiso Trust [18].

Silicosis risk in the working environment is a function of duration of exposure, rate of exposure, time since first exposure (‘latency’), age, the airborne concentration of silica dust, and lack of respiratory protective equipment [19]. Leger was one of the first commentators in the period of interest to call attention to an upward trend in silicosis incidence among migrant black workers in the South African mining industry, drawing on compensation data and company reports of silicosis [20]. He attributed the trend after 1975 to ‘stabilisation’ of the migrant workforce, that is, a series of recruitment measures introduced by the gold mining industry to convert migrant miner employment from intermittent short-term contracts with frequent gaps in between to that of continuous service with miners returning annually to the same mine. Leger showed a sharp rise in duration of service and age of employed miners over the decade of the 1980s, a trend continued and noted in the later studies cited above [8,10].

Unprecedented access to the employment database of a large South African gold mining company going back more than four decades provided us with an opportunity to examine changes in patterns of cumulative employment in detail. The broad aim of this study was to demonstrate how major changes in recruitment practice, driven by changes in the global and local political and economic environments, provided a basis for this silicosis epidemic. These changes included the influx of a large number of novice miners, incentives to migrant miners to become ‘career’ miners, and retention in service of miners with silicosis and TB.

Our specific analytic objective was to demonstrate the sustained rise in mean cumulative employment at exit among black migrant workers as the basis for the rising silicosis risk over the 45-year span of this database. This information comes at a time of renewed interest in silicosis risk, as the two compensation programmes described above seek to manage the task of finding and examining a population estimated in the hundreds of thousands—one neglected for 120 years.

## Methods

### The database

The database was recently made available by one of South Africa’s largest gold mining companies as part of efforts to support the functioning of the statutory compensation system and the class-action settlement [17,18]. The database analysed was restricted to 300,774 ex-miners who had worked at this company and exited between 1973 and 2018. The study was approved by the Human Research Ethics Committee, Faculty of Health Sciences, University of Cape Town (ref. 092/2021). As the study was based on anonymised administrative data, the need for individual consent was waived.

The variables available from the database are set out in Table 1. All contracts have information on start and end dates. Individual miners were identified with a unique key assigned at commencement by TEBA (PTY) Ltd, formerly the Employment Bureau of Africa and historically the primary gold miner recruiting and registration agency [3]. Reliable data on race and occupation were not available. However, black miners have been shown to comprise between 88% and 95% of the workforce at different times [3], and it is estimated that 80%–90% of employees were in dust-exposed occupations. Migrant status was also not recorded in the database, but almost all cross-border workers and the large majority of South African workers can be assumed to be migrants.

**Table 1:** Variables used in this study.


VARIABLE ON DATABASE	COMMENT OR FURTHER DESCRIPTION	DERIVED VARIABLES (FOR EACH MINER)

Unique key	TEBA (PTY) Ltd randomly assigned industry number (not personal ID)	–

Country of origin	Country or office at which recruited or registered for a given contract	Country of origin*

Contract start date		Number of contractsStart date of first contractStart date of last contract

Contract termination date		End date of first contractEnd date of last contract

Contract duration (days)	Difference in days between contract termination date and contract start date plus 1	Cumulative employment across all contracts in days or years Contract duration years where 1 year = 365.2425 days

Date of birth		Age at start of first contractAge at end of last contract


* Miners recruited from nominally independent “homelands” in the apartheid era are counted as South African. A small number of miners from Lesotho may have changed country of origin during this period.

### Statistical analysis

All miners were assigned a value for the calendar date at which their final observed contract terminated, with cumulative employment calculated by summing all the days worked on all contracts to that point. This allowed the analysis of differences in the probability distributions of cumulative employment among miners whose final contract terminated in different periods.

Means with standard deviations and medians with interquartile ranges, medians, ranges, frequency proportions, and percentiles were calculated for variables of interest for the entire observed period from 1973 to 2018. Mean and median cumulative employment at exit were calculated for each exit year over the observed period. We further calculated the proportion of miners exiting each year with at least 10, 15, or 20 years of employment. These are typical thresholds cited for the radiological appearance of chronic silicosis [21,22].

To investigate changing trends through the observed period, piecewise linear regression was used, as described by Muggeo [23]. We aimed to estimate segmented linear relationships between the calendar date of last contract and the total length of service by finding both the slope of each segment and breakpoint dates between the segments. The number of breakpoints was determined using a stepwise iterative approach, selecting the one increasing the number of breakpoints and calculating the adjusted-R^2^ of the piecewise linear model with each step. We stopped the process once the adjusted-R^2^ value had stabilised, with additional iterations providing less than 1% improvement in adjusted-R^2^.

Finally, a stratified analysis was carried out comparing South African miners with ‘cross-border miners’ from Botswana, Eswatini, Lesotho, and Mozambique, currently the major labour-sending neighbouring countries.

## Results

### Overall

Table 2 gives the contract characteristics of the sample of underground miners. Of the 300,774 miners, 55% (165,949) were of South African origin, 30% (90,390) were recruited from neighbouring countries, and the remaining 15% were unspecified. Since cross-border miners have to pass through South African immigration procedures which would probably be recorded in the database, it is likely that the miners in the unspecified group are of South African origin, in which case the ‘local’ proportion of the total would be 70%, consistent with that of the industry as a whole [3].

**Table 2:** Contract characteristics of employees on the database of a large gold mining company, 1973–2018 (n = 300,774).


VARIABLE	MEAN	SD	MEDIAN	IQR

Age at first contract (years)	23.3	6.2	21.6	19.7, 25.0

Age at the start of the last contract (years)	40.3	11.7	40.4	30.4, 49.9

Age at the end of the last contract (years)	42.5	11.5	42.4	33.0, 51.8

Calendar year of first contract	1980	10.7	1981	1974, 1986

Calendar year of last contract	1997	(9.4)	1996	1989, 2003

Cumulative employment at end of final contract (years)	13.8	(8.9)	12.7	6.9, 19.4


SD: standard deviation; IQR: interquartile range.

### Trends

**Figure 1:**
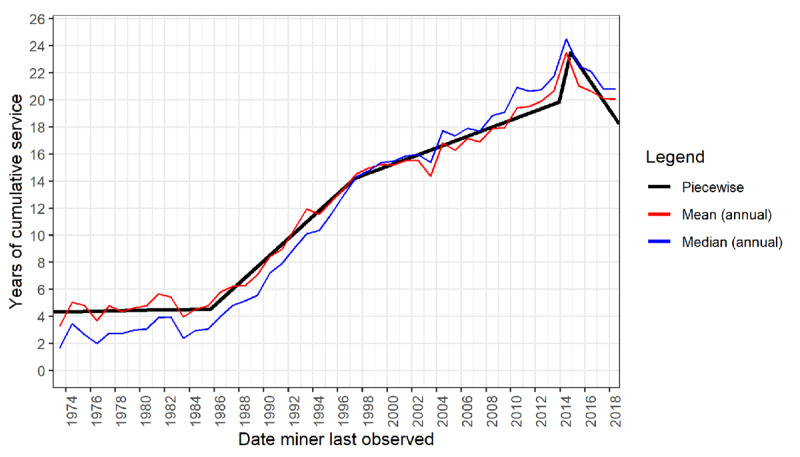
Years of cumulative employment* of employees of a major gold mining company, by final contract end date, 1973–2018**. * Annual mean and median values calculated by calendar year of last observation. ** Includes service at other gold mining companies.

Table S1 (Supporting Information) tabulates the mean and median cumulative employment at exit by year for the period 1973–2018. Table S2 groups the data into five-year intervals, with the addition of selected percentiles. Figure S1 presents the annual data graphically, including percentiles. While the overall trend is upwards, stepwise linear regression identified five phases of change in the cumulative employment duration. Figure 1 and Table 3 present the piecewise regression model characteristics for the whole cohort.

**Table 3:** Piecewise linear regression slope coefficients with four breakpoints*.


DATE RANGE	NUMBER OF WORKERS	BREAKPOINT (95% CI)	SLOPE (YEARS/CALENDAR YEAR) (95% CI)

1973-01-01 to 1985-09-16	15,673	1985-09-16 ± 84 days	0.02 (–0.04, 0.07)

1985-09-17 to 1997-05-11	130,522	1997-05-11 ± 75 days	0.83 (0.77, 0.89)

1997-05-12 to 2013-11-30	126,496	2013-11-30 ± 49 days	0.34 (0.33, 0.36)

2013-12-01 to 2014-11-06	8,208	2014-11-06 ± 21 days	3.93 (3.26, 4.60)

2014-11-07 to 2018-12-31	19,875	–	–1.35 (–2.02, –0.67)


* Adjusted R^2^ of model = 0.283; CI: Confidence interval.

The first phase, from 1973 to 1985, shows no significant change in cumulative employment with a fluctuation around the mean of approximately four years. In the second phase, from 1985 to 1997, cumulative employment increases at 0.83 (95% confidence interval [CI] 0.77, 0.89) years per calendar year, reaching a mean annual duration of 14.6 years in 1997. The third phase, from 1997 to 2013, shows a slower rate of increase of 0.34 (95% CI 0.33, 0.36) years per calendar year, reaching a mean annual cumulative employment of 20.7 years.

In the fourth phase, from 2013 to 2014, there is a rapid increase over a one-year period, yielding a ‘peak’ mean duration of employment of 23.5 years. Finally, there is a decline in the last period observed, 2014–2018, with mean cumulative employment of 20.1 years. Overall, ignoring fluctuations, these data show a fivefold increase in cumulative employment at exit over the period 1973–2018.

**Figure 2:**
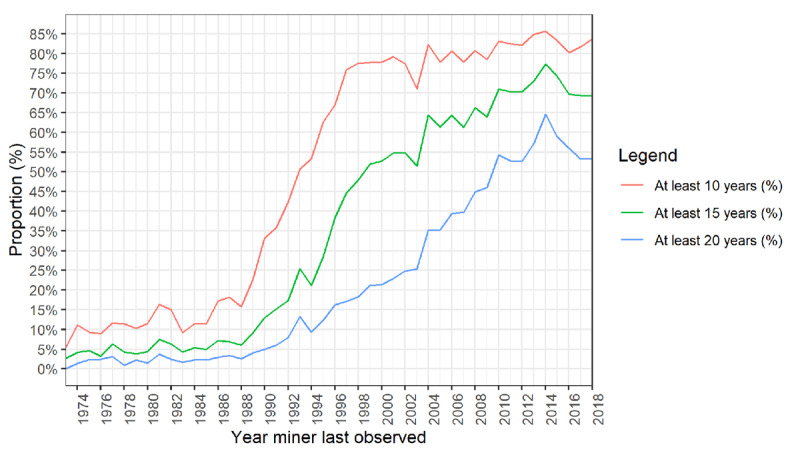
Proportion of annual exit cohort with ≥10, 15, or 20 years cumulative employment, by final contract end date, 1973–2018.

Figure 2 shows the increase in the proportion of gold miners with cumulative employment at ≥ 10, 15, and 20 years’ duration. There is a steady rise in the proportion in each category from 1987 continuing unabated until 2014. The ≥15-year stratum reaches a peak proportion of over 77% of the annual exit cohort in 2014, while the ≥20-year stratum reaches 65% in that year.

### South African versus cross-border miners

**Figure 3:**
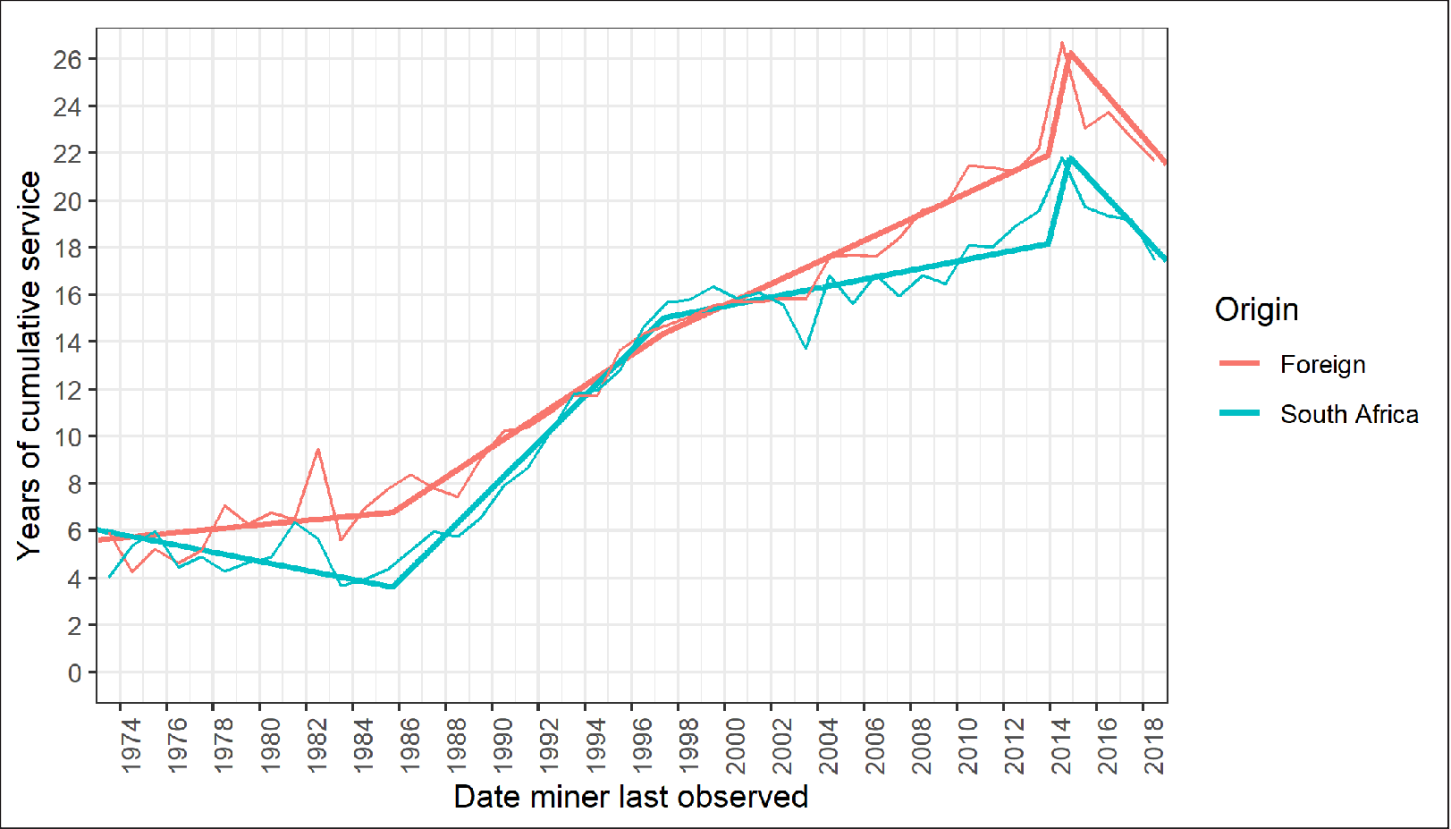
Years of cumulative employment by final contract end date: miners of South African origin versus those from neighbouring countries,* 1973–2018**. * Botswana, Eswatini, Lesotho, Mozambique. ** Piecewise linear regression (thick lines) and annualised mean (thin lines), using the same breakpoints as in Figure 1.

Finally, Figure 3 compares the trends in cumulative employment between South African and cross-border miners. Over most of this period, cross-border miners accumulated greater service than South Africans at exit. In the period 1973–1996, this gap widened and then closed. From 2000 to 2018, it widened again, then it stabilised at a mean excess of four years. Figure S2 (Supporting Information) disaggregates these curves by country of origin.

## Discussion

In 1996, a South African commission of inquiry into health and safety in the mining industry noted that no direct measure of mine service of the mining workforce as a whole existed and that much information was held in confidence by management [24]. This article contains the first detailed analysis of long-term trends in cumulative duration of employment among miners at one company at the time of exit. It supports the contention of previous commentators that a major driver of the increase in silicosis in the late 20th century, noted among black miners from company reports, compensation data, and autopsy analysis, was the year-on-year increase in cumulative employment and by inference in cumulative exposure to silica dust [8,20].

### Dust trends

Cumulative dust exposure is the product of duration of exposure and average dust concentration. The above-mentioned commission of inquiry noted that in 1996 dust levels had remained ‘roughly [the] same’ since the 1940s [24]. Reliable airborne respirable silica measurements using modern gravimetric methods from which secular trends over our period of interest, 1973, to date could be calculated are lacking, especially given the late changeover from konimetric to gravimetric dust sampling in the early 1990s [25,26]. A summary of over 26,000 respirable silica concentrations on South African gold mines measured between 1995 and 1997 showed that 13% exceeded the South African mining occupational exposure limit (OEL) for respirable silica of 0.1 mg.m^3^. Of 48 mines, only 8 were fully compliant with this level, with the remaining having one or more measurements exceeding the limit [27]. The OEL of 0.1 mg/m^3^, which is intended to protect 95% of the workforce over a lifetime of work exposure, has itself been shown to be non-protective in the South African gold mining industry [18,28]. Therefore, while there is no evidence of a sustained rise in silica dust concentrations over the period of interest, consistent exposure to hazardous concentrations serves as the ‘baseline’ condition by which rising cumulative employment acts to elevate silica risk.

### Change in employment patterns

A sharp rise and then fall in the numbers employed in the South African gold mining industry in the late 20th century [3] is reflected in the experience of the company studied in this report, where the numbers employed in 1973, 1988, and 2018 were approximately 61,000, 240,000, and 43,000, respectively [29]. Contributing to this rise and fall were a number of internal and external forces acting on the industry from the 1970s, resulting in changes in employment practice and patterns. These changes were commonly referred to as ‘stabilisation’, as they had the intention (and effect) of reducing labour turnover and recruitment uncertainties which had up that time characterised the migrant labour system [1,20]. Globally, there was a rapid and sustained increase in the dollar price of gold over the course of the 1970s, from US $36/ounce to US $613/ounce, after the United States abandoned the convertibility of the dollar into gold in 1971 [30]. Regional factors included the occurrence of acute labour shortages following the withdrawal of workers from Malawi and restrictions on recruitment from Mozambique in 1976 after independence from Portugal was attained [1].

In response to the threat of labour shortages, the mining industry embarked on rapid recruitment by escalating the engagement of novices from the Eastern Cape region of South Africa, for whom mining employment had become relatively more attractive as a result of the decline in manufacturing jobs [1]. This was accompanied by a significant rise in the wages of black miners, which had not risen in real terms for most of the century [31], and the acceptance by the industry of the practice of allowing black production workers to take up higher skilled jobs and to be trained accordingly [1]. A system of incentives in the form of contract security and special payments for returning to the same job, combined with penalties for failing to meet contract requirements, was used to retain migrant employees as ‘career miners’ [1].

Immediate consequences of these various developments were the entry of a large number of novices of South African origin with fewer cross-border recruits and a rapid increase in the proportion of migrant workers returning annually to the same companies. While labour shortages abated by the end of the 1980s, this strategy continued in the form of last in, first out (LIFO) retrenchment policies and preferential re-employment of skilled, older, longer service workers as the gold mining industry entered a long period of employment decline after 1990 (F. Baleni, Dr JPL Bezuidenhout, personal communication).

A further factor was a change in practice, given legal sanction by the statutory certifying agency, the Medical Bureau of Occupational Diseases (MBOD), in the late 1970s and 1980s to allow miners with ‘first degree’ silicosis or treated TB to return to underground work [2,32]. This was contrary to the long-standing legal requirement in the mining industry barring such workers or work seekers from underground or equivalent ‘risk’ work. The year-on-year impact of this change in practice is difficult to disentangle from other changes, especially as there is no documented follow-up of the impact of these changes on the health of miners and ex-miners [2]. The most likely effect would have been a rise in silicosis prevalence in working miners during the period after the practice was introduced in the late 1970s and 1980s. This rise would have reached a limit as new diagnoses approached an equilibrium with the exit of miners with silicosis [10].

Historians, notably McCulloch [2] and Marks [33], have argued that it is incorrect to view long service and silicosis in black migrant miners as a uniquely late 20th-century phenomenon and to assert that short intermittent service had ‘protected’ such miners from silicosis prior to the 1970s/1980s. Citing autopsy findings and reports of medical observers and official investigations, they suggested that silicosis was always prevalent in black migrant miners but obscured by a number of factors. These included cursory medical examinations at recruitment, repatriation of affected or sick workers without follow-up, lack of access of black miners to compensation and statutory autopsy once they had left the mines, and, in general, an exaggeration of success in controlling the disease based only on white workers’ experience [2,33].

An exception with respect to cumulative service is the work of First in Mozambique in 1977 [34]. First interviewed a sample of 145 gold miners in Inhambane province who had worked on the South African gold mines. Between 40% and 43% of their working lives had been spent on the mines. This proportion was the same in all age groups, suggesting no secular trend upwards or downwards. Among a further 226 miners (average age 51 years) from 10 other provinces in the process of returning to mine employment, the average cumulative employment was 10 years. This figure is much higher than the average of 1 to 4 years for Mozambiquan miners exiting between 1973 and 1978 calculated from the database analysed for this report (Figure S2).

In two later rural surveys of ex-miners who had worked on the gold mines in the 1980s or earlier, one in Botswana and one in the Transkei region of South Africa, mean duration of employment was 14 years and 12.3 years, respectively [11], considerably higher than the mean values in Figure S2 of this report and closer to the 90th percentile (Table S2 and Figure S1).

These studies do not identify the mines where the participants had worked, and it is possible that recruitment practice varied among different companies in the early years of the changes in recruitment practice described above. The turnover associated with the large-scale recruitment of novices is likely to have diluted the mean cumulative employment at exit of South African workers [1]. However, the early phase of the data reported in this study, particularly those of cross-border miners, should be treated with caution. Previous analysis of industry-wide recruitment showed that digitisation of the TEBA database was incomplete during the 1970s and underestimated employment relative to that reported by government [3]. This undercounting may have affected cross-border workers in particular, whose share of the workforce at the company described here declined from 51% in 1973 to 30.6% in 1985 [29].

In summary, substantial cumulative service and significant silicosis risk are likely to have been features of the migrant gold mining population prior to the period of this study. The late 20th-century shift in recruitment and employment practice would therefore have brought to light phenomena previously hidden by lack of surveillance of departing and ex-migrant miners. However, the deficiencies in the historical record until the 1990s make it difficult to quantify such cumulative service and associated silicosis risk. Further research is needed to fill this gap and to inform interpretation of the results of this study.

### Strengths and limitations

A prime consideration for study validity is the accuracy of employment duration on the company database on which this analysis is based. The company itself grew from a localised base in 1973 by incorporating other gold mining companies so that many of its employees would have served in several companies, as was the case across the industry in earlier decades. Additional factors influencing the accuracy of the data might be incomplete digitisation of records by the recruiting agency TEBA prior to the 1980s or the incomplete registration of miners bypassing TEBA and signing on directly at the mine or of miners employed via labour brokers or by subcontractor companies [1,3].

The database used for this research was provided by the company for use by the statutory compensation agency, MBOD, and the civil Tshiamiso Trust. It includes miners taken on through acquisitions of other companies and includes service at other mines and time worked as subcontractors. It has been updated with employment information obtained from miner pension and provident funds and other insurance agencies, banks, the Department of Home Affairs, and employment-related documents, such as training certificates and licences (Dr Z. Eloff, personal communication). However, some uncaptured service is probable. This is more likely to have occurred during 1970s, resulting in a lower observed baseline and an exaggerated ‘take-off’ of the cumulative employment curve.

With respect to generalisability, the South African mining industry has been dominated by relatively few large companies, with interlocking and changing ownership. It is estimated that the Tshiamiso Trust settlement by six companies covered approximately 80% of gold mine employment over the period identified for settlement, that is, 1965–2018 (Dr Z. Eloff, personal communication). Recruitment policies and practices were highly centralised via TEBA and the employer association, the Chamber of Mines (now the Minerals Council South Africa). In a study of silicosis prevalence between 2004 and 2009 covering three of the large gold mining companies, no statistically significant difference in silicosis prevalence was found among these companies [21]. It is therefore likely that an analysis of the employment of other companies would have found similar employment trends.

## Conclusions

Drawing on newly available data, we were able to demonstrate the increase in cumulative employment at exit among black migrant workers between 1987 and 2016 and the increase in the proportion of miners working 10,15, or 20 years or more. Against the background of non-protective silica dust concentrations in the gold mines, this increase in cumulative exposure to inhaled silica dust is likely to be the primary explanation for the sharp rise in silicosis observed in a new cohort of working miners in the late 20th and early 21st centuries.

The purpose of this study was not to study the association between cumulative employment and silicosis, which is well established [19]. Besides confirming the experience of a particular cohort, the findings fill an important gap in our understanding of the current risk of silicosis borne by this cohort. This is of public health importance, as the current programmes for redress do not have reliable estimates of burden of disease. While autopsy surveillance has provided unique information on trends, silicosis at autopsy is not equivalent to radiological silicosis. The record in living miners is sparse, with the last systematic workforce survey being from 2006 to 2009 [17,21], followed by a report of compensation examinations of ex-miners from Lesotho in 2017–2019 [35]. Past annual numbers of compensation claims are not a reliable source owing to poor access by ex-miners and the historical dysfunction of the statutory compensation system [11,14,15,16].

There were a number of changes in the recruitment practices of migrant labour in the South African gold mining industry operating from the 1970s that produced the epidemic of silicosis among active gold miners by the 1990s and early 2000s. These changes were augmented by changes in informal and statutory medical exclusion policies that allowed miners with silicosis or treated TB, previously required to be excluded by law, to rejoin or remain in service.

While all former miners have suffered from lack of surveillance, medical follow-up, and access to compensation and statutory post-mortem examinations, cross-border miners are in a worse position than South African miners [29,36]. For most of this period, cross-border miners have had higher cumulative employment at exit than South African miners, the recent mean excess being four years. The implication is an even larger silicosis risk burden, as suggested by the findings of the recent study of ex-miners in Lesotho [35]. As the population of cross-border ex-miners ages and dies, this consideration lends further urgency to the mandate of the statutory agency and the Tshiamiso Trust, and particularly so given the difficulties of fulfilling this mandate in neighbouring countries [29,36].

The findings of this analysis also illustrate a broader phenomenon. It is important to understand the influence of economic and sociopolitical conditions, such as a change in the gold price, recruitment policies, and discriminatory access to medical examination and surveillance systems, to properly understand changes over time in the occurrence of occupational disease and injury and its impact on society. These are population-level or contextual variables rather than individual. This approach has application to other occupational injury and disease risk settings globally [37].

## Additional File

The additional file for this article can be found as follows:

10.5334/aogh.4059.s1 Supporting Information. Annals Of Global Health.

## References

[1] Crush J, Jeeves A, Yudelman D. South Africa’s Labor Empire. A History of Black Migrancy to the Gold Mines. Witwatersrand University Press; 1991.

[2] McCulloch J. South Africa’s Gold Mines and the Politics of Silicosis. James Currey; 2012.

[3] Ehrlich RI, Montgomery A, Akugizibwe P, Gonsalves G. Health implications of changing trends in the origins and characteristics of mineworkers in South Africa, 1973–2012. BMC Public Health. 2017; 18(1): 93. DOI: 10.1186/s12889-017-4640-x 28774280 PMC5543439

[4] Rees D, Murray J, Nelson G, Sonnenberg P. Oscillating migration and the epidemics of silicosis, tuberculosis and HIV infection in South African gold miners. Am J Ind Med. 2010; 53(4): 398–404. DOI: 10.1002/ajim.20716 19565628

[5] Park HH, Girdler-Brown BV, Churchyard GJ, White NW, Ehrlich RI. Incidence of tuberculosis and HIV and progression of silicosis and lung function impairment among former Basotho gold miners. Am J Ind Med. 2009; 52(12): 901–908. DOI: 10.1002/ajim.20767 19882740

[6] Ehrlich R, Akugizibwe P, Siegfried N, Rees D. Silica exposure, silicosis and tuberculosis—a systematic review. BMC Public Health. 2021; 21: 953. DOI: 10.1186/s12889-021-10711-1 34016067 PMC8136154

[7] Corbett EL, Churchyard GJ, Clayton TC, et al. HIV infection and silicosis: the impact of two potent risk factors on the incidence of mycobacterial disease in South African miners. AIDS. 2000; 14(17): 2759–2768. DOI: 10.1097/00002030-200012010-00016 11125895

[8] Nelson G, Girdler-Brown B, Ndlovu N, Murray J. Three decades of silicosis: disease trends at autopsy in South African gold miners. Environ Health Perspect. 2010; 118: 421–426. DOI: 10.1289/ehp.0900918 20194070 PMC2854773

[9] Cowie RL, van Schalkwyk MG. The prevalence of silicosis in Orange Free State gold miners. J Occup Med. 1988; 29(1): 44–46. 3819884

[10] Knight D, Ehrlich RI, Fielding K, Jeffery H, Grant A, Churchyard G. Trends in silicosis prevalence and the healthy worker effect among active gold miners in South Africa. BMC Public Health. 2015; 15: 1258. DOI: 10.1186/s12889-015-2566-8 26686997 PMC4684919

[11] White NW, Steen TW, Trapido ASM. Occupational lung diseases among former goldminers in two labour sending areas. S Afr Med J. 2001; 91(7): 599–604. https://www.ajol.info/index.php/samj/article/view/140041. 11544979

[12] Churchyard GJ, Ehrlich R, teWaterNaude JM, et al. Silicosis prevalence and exposure-response relations in South African goldmines. Occup Environ Med. 2004; 61: 811–816. DOI: 10.1136/oem.2003.010967 15377766 PMC1740677

[13] Davies JC. Sound an alarm! S Afr Med J. 1994; 84: 133–134. DOI: 10.1016/0167-7187(94)90032-9 7740346

[14] Davies JC. Silicosis and tuberculosis among South African goldminers—an overview of recent studies and current issues. S Afr Med J. 2001; 91(7): 562–566. 11544967

[15] Ehrlich RI. A century of miners’ compensation in South Africa. Am J Ind Med. 2012; 55(6): 560–569. DOI: 10.1002/ajim.22030 22431163

[16] Kistnasamy B, Yassi A, Yu J, et al. Tackling injustices of occupational lung disease acquired in South African mines: recent developments and ongoing challenges. Global Health. 2018; 14(1): 60. DOI: 10.1186/s12992-018-0376-3 29954399 PMC6022447

[17] Minerals Council South Africa. Joint effort to improve the occupational lung disease compensation system. Fact sheet. February 2020. Accessed November 27, 2022. https://www.mineralscouncil.org.za/search-results?q=compensation.

[18] Tshiamiso Trust. Origins of the Trust. Accessed November 27, 2022. https://www.tshiamisotrust.com/about/origins/.

[19] Sun Y, Bochmann F, Morfeld P, et al. Change of exposure response over time and long-term risk of silicosis among a cohort of Chinese pottery workers. Int J Environ Res Public Health. 2011; 8(7): 2923–2936. DOI: 10.3390/ijerph8072923 21845166 PMC3155337

[20] Leger JP. Occupational disease in South African mines—a neglected epidemic. S Afr Med J. 1992: 76: 557–561. 1738906

[21] Knight D, Ehrlich R, Cois, A, Fielding K, Grant A, Churchyard G. Predictors of silicosis in an industry wide study of working gold miners. BMC Public Health. 2020; 20: 829. DOI: 10.1186/s12889-020-08876-2 32487111 PMC7268682

[22] Álvarez RF, González CM, Martínez AQ, Pérez JJB, Fernández LC, Fernández AP. Guidelines for the diagnosis and monitoring of silicosis. Arch Bronconeumol. 2015; 51(2): 86–93. DOI: 10.1016/j.arbr.2014.07.002 25479706

[23] Muggeo VMR. Estimating regression models with unknown break-points. Stat Med. 2003; 22(19): 3055–3071. DOI: 10.1002/sim.1545 12973787

[24] Republic of South Africa (Department of Minerals and Energy). Report of the Commission of Inquiry into Health and Safety in the Mining Industry. Vol. 1. Dept of Minerals and Energy; 1995. Accessed December 24, 2022. https://www.dmr.gov.za/Portals/0/Resource%20Center/Reports%20and%20Other%20Documents/2003_Leon%20Commission_Volume%201.pdf?ver=2018-03-13-020431-270.

[25] Unsted AD. Personal gravimetric dust sampling and risk assessment. Johannesburg: Safety in Mines Research Advisory Committee (SIMRAC). Project GAP046; March 1996. Accessed December 24, 2022. https://researchspace.csir.co.za/dspace/handle/10204/1680.

[26] Brouwer DH, Rees D. Can the South African milestones for reducing exposure to respirable crystalline silica and silicosis be achieved and reliably monitored? Front Public Health. 2020; 8: 107. DOI: 10.3389/fpubh.2020.00107 32318535 PMC7154115

[27] National Centre for Occupational Health (NCOH). A Report on Occupational Health Indicators for South Africa Part II. Report no. 1/99. NCOH. 1999; 16–24.

[28] Hnizdo E, Sluis-Cremer G. Risk of silicosis in a cohort of white South African gold miners. Am J Ind Med. 1993; 24: 447–457. DOI: 10.1002/ajim.4700240409 8250063

[29] Ehrlich R, Barker S, Tsang VWL, Kistnasamy B, Yassi A. Access of migrant gold miners to workers’ compensation for occupational lung disease: quantifying a legacy of injustice. J Migr Health. 2021; 4: 100065. DOI: 10.1016/j.jmh.2021.100065 34729543 PMC8546409

[30] Kane-Berman J. Mining in SA: Then, now, and into the future—IRR. Politicsweb; February 15, 2017. https://www.politicsweb.co.za/documents/mining-in-sa-then-now-and-into-the-future--irr.

[31] Wilson F. Minerals and migrants: how the mining industry has shaped South Africa. Daedalus. 2001; 30(1): 99–121. https://www.jstor.org/stable/20027681.

[32] Cowie RL. Silicosis, Pulmonary Dysfunction and Respiratory Symptoms in South African Gold Miners. Dissertation. University of Cape Town; 1987: 1–188.

[33] Marks S. The silent scourge? Silicosis, respiratory disease and gold-mining in South Africa. Journal of Ethnic and Migration Studies. 2006; 32(4): 569–589. DOI: 10.1080/13691830600609975

[34] First R. Black Gold: The Mozambiquan Miner, Proletarian and Peasant. Harvester Press; 1983.

[35] Maboso BM, Moyo DM, Muteba KM, et al. Burden of disease among Basotho ex-miners in a large out-reach medical assessment programme. Occup Health Southern Afr. 2020; 26(4): 145–152. https://hdl.handle.net/10520/ejc-ohsa-v26-n4-a2.

[36] Aids and Rights Alliance for Southern Africa (ARASA). The mining sector, tuberculosis and migrant labour in Southern Africa; 2008. Accessed December 6, 2022. http://catalogue.safaids.net/sites/default/files/publications/The%20Mining%20Sector,%20Tuberculosis%20and%20Migrant%20Labour%20in%20Southern%20Africa.pdf.

[37] Fair/Square. Qatar. Accessed December 6, 2022. https://fairsq.org/category/qatar/page/2/.

