# Supplementary material for: Mining Migrant Worker Recruitment Policy and the Production of a Silicosis Epidemic in Late 20th-Century Southern Africa
Source: Ann Glob Health. 2023 Mar 27;89(1):25. doi: 10.5334/aogh.4059 (PMC10064917; doi:10.5334/aogh.4059)

SUPPORTING INFORMATION: ANNALS OF GLOBAL HEALTH

Migrant worker recruitment policy and the production of a silicosis epidemic in late 20th Century Southern Africa

Rodney Ehrlich, Stephen Barker, Alex Montgomery, Barry Kistnasamy, Annalee Yassi

Table S1 Annual mean and median cumulative employment (years), by final contract end date, 1973-2018

Year	Workers	Mean (95% CI)	Median (IQR)
1973	75	3.27 (2.42, 4.13)	1.66 (1.00, 4.02)
1974	72	5.05 (3.80, 6.30)	3.46 (1.45, 6.56)
1975	86	4.82 (3.57, 6.06)	2.68 (1.00, 6.33)
1976	124	3.69 (2.87, 4.52)	2.00 (1.00, 4.66)
1977	190	4.79 (4.01, 5.56)	2.77 (1.12, 6.39)
1978	211	4.37 (3.73, 5.01)	2.74 (1.14, 5.42)
1979	263	4.62 (3.95, 5.29)	3.00 (1.65, 5.42)
1980	277	4.80 (4.23, 5.37)	3.07 (1.99, 6.22)
1981	269	5.66 (4.96, 6.36)	3.93 (2.00, 6.85)
1982	413	5.45 (4.92, 5.98)	3.99 (1.88, 7.14)
1983	2692	3.97 (3.79, 4.15)	2.41 (0.80, 5.36)
1984	6177	4.53 (4.41, 4.66)	2.97 (1.00, 6.00)
1985	6481	4.78 (4.66, 4.91)	3.07 (1.21, 6.65)
1986	5065	5.80 (5.65, 5.95)	4.00 (1.90, 7.99)
1987	6102	6.23 (6.09, 6.37)	4.83 (2.34, 8.44)
1988	5906	6.29 (6.16, 6.43)	5.16 (2.81, 8.35)
1989	6506	7.07 (6.93, 7.21)	5.56 (3.00, 9.67)
1990	7097	8.42 (8.27, 8.56)	7.23 (3.75, 11.65)
1991	8632	8.97 (8.84, 9.10)	7.92 (4.12, 12.17)
1992	16067	10.50 (10.40, 10.60)	9.09 (6.29, 12.99)
1993	9996	11.95 (11.80, 12.11)	10.12 (6.67, 15.12)
1994	25847	11.56 (11.47, 11.64)	10.39 (7.32, 14.14)
1995	22680	12.51 (12.42, 12.61)	11.61 (7.91, 15.79)
1996	10928	13.49 (13.34, 13.63)	13.01 (8.23, 17.72)
1997	16700	14.55 (14.44, 14.66)	14.30 (10.18, 18.19)
1998	17941	14.95 (14.85, 15.05)	14.77 (10.58, 18.62)
1999	9160	15.26 (15.11, 15.41)	15.39 (10.72, 19.27)
2000	7800	15.19 (15.03, 15.34)	15.47 (10.79, 19.26)
2001	8131	15.53 (15.38, 15.67)	15.86 (11.13, 19.63)
2002	6141	15.54 (15.37, 15.72)	15.97 (10.79, 19.99)
2003	5898	14.36 (14.15, 14.57)	15.37 (8.74, 20.05)
2004	8062	16.84 (16.68, 17.00)	17.74 (12.41, 21.61)
2005	5584	16.29 (16.08, 16.49)	17.35 (11.08, 21.73)
2006	4349	17.17 (16.95, 17.39)	17.91 (12.07, 22.51)
2007	5946	16.90 (16.69, 17.11)	17.70 (10.86, 22.97)
2008	6889	17.88 (17.69, 18.08)	18.85 (12.13, 23.95)

2009	7651	17.94 (17.74, 18.15)	19.10 (11.40, 24.39)
2010	6621	19.42 (19.20, 19.63)	20.95 (13.46, 25.76)
2011	4859	19.53 (19.28, 19.78)	20.65 (13.21, 26.14)
2012	4971	19.91 (19.65, 20.18)	20.75 (13.36, 26.50)
2013	4030	20.68 (20.41, 20.96)	21.78 (14.33, 27.30)
2014	11611	23.49 (23.29, 23.69)	24.52 (15.93, 30.62)
2015	5349	21.06 (20.81, 21.31)	22.51 (14.54, 28.03)
2016	4123	20.66 (20.36, 20.96)	22.13 (12.59, 28.47)
2017	6078	20.13 (19.90, 20.36)	20.79 (12.54, 27.31)
2018	724	20.07 (19.41, 20.72)	20.81 (12.87, 27.26)

CI: confidence interval. IQR: interquartile range

Table S2 Distribution of cumulative employment (years) by final contract end date, grouped into 5-year periods: selected percentiles and mean,1973-2018

**Period**	**10th percentile**	**25th percentile**	**50th percentile**	**75th percentile**	**90th percentile**	**Mean**
1973-1977	0.58	1.00	2.30	5.58	9.82	4.37 (3.94, 4.80)
1978-1982	1.00	1.70	3.37	6.51	11.43	5.05 (4.77, 5.33)
1983-1987	0.54	1.40	3.53	7.00	11.87	5.17 (5.11, 5.23)
1988-1992	2.00	4.34	7.64	11.68	16.69	8.80 (8.74, 8.86)
1993-1997	4.39	7.86	11.67	16.16	21.70	12.68 (12.63, 12.73)
1998-2002	6.00	10.75	15.26	19.21	23.09	15.22 (15.15, 15.28)
2003-2007	4.72	11.09	17.22	21.75	25.27	16.31 (16.22, 16.39)
2008-2012	5.35	12.56	19.90	25.16	29.36	18.81 (18.71, 18.91)
2013-2017	7.15	14.34	22.76	28.86	33.41	21.68 (21.57, 21.79)
2018	7.57	12.87	20.81	27.26	30.94	20.07 (19.41, 20.72)
**Total**	2.79	6.94	12.67	19.36	25.96	13.75 (13.72, 13.78)

Figure S1 Distribution of cumulative employment (years) by final contract end date: selected percentiles and mean, 1973-2018

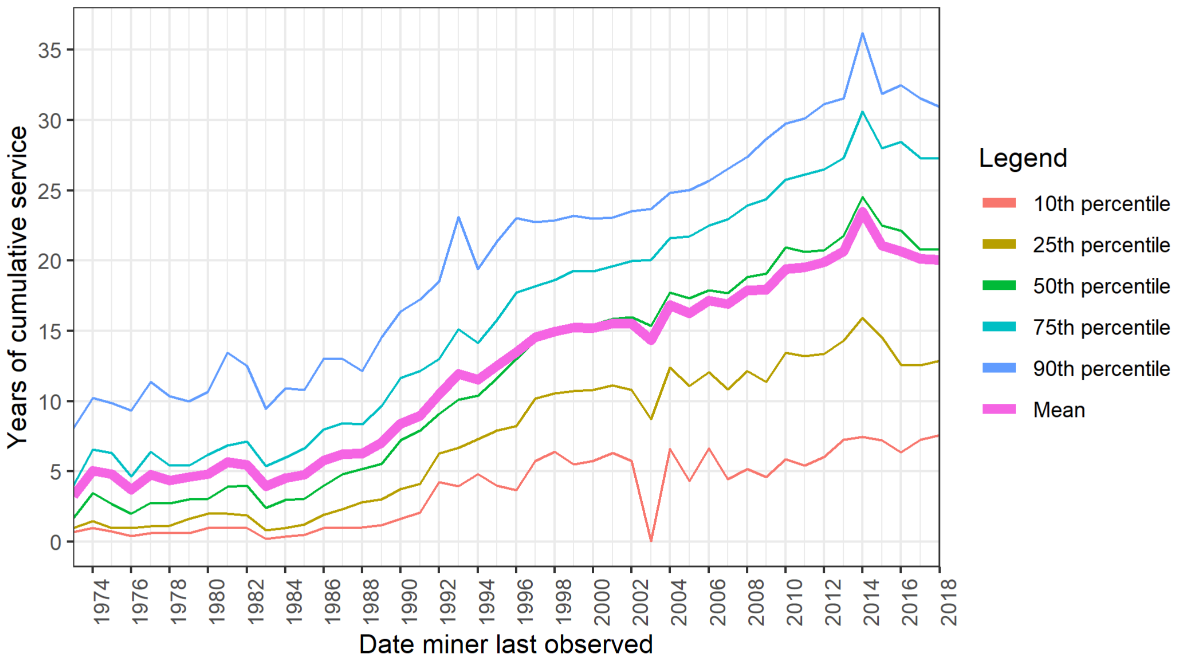

Figure S2 Cumulative employment (years) by final contract end date, by country of origin, 1973-2018

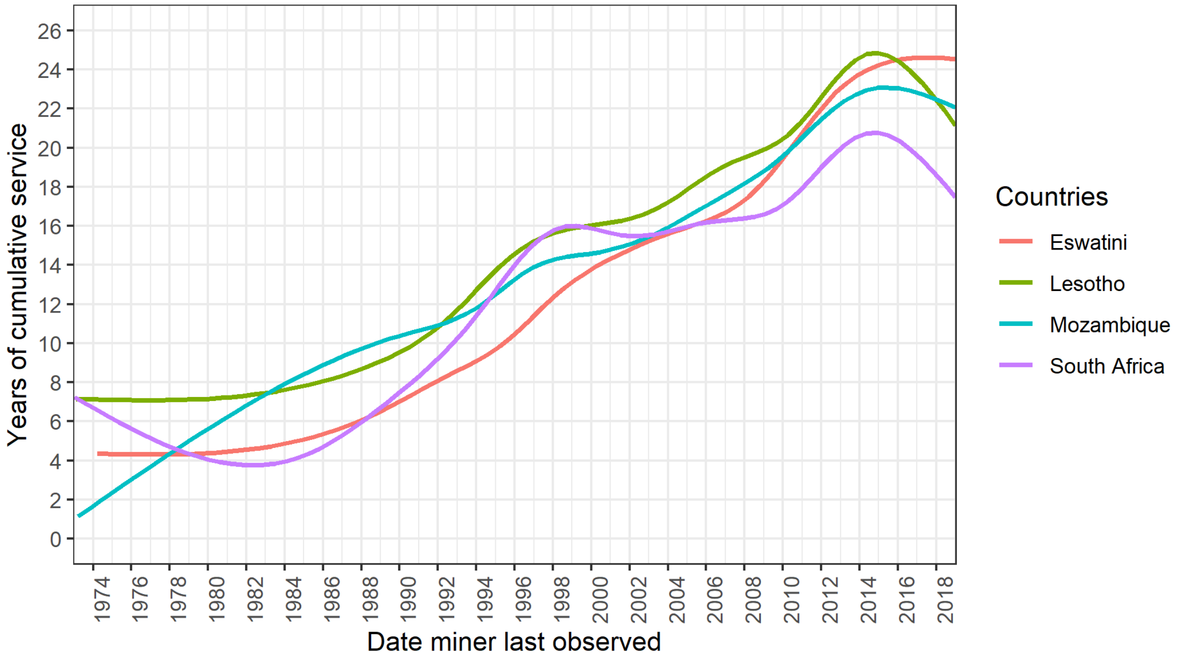

Supplement: Supporting Information. — Annals Of Global Health. [file agh-89-1-4059-s1.pdf]
